# mHealth-Supported Gender- and Culturally Sensitive Weight Loss Intervention for Hispanic Men With Overweight and Obesity: Single-Arm Pilot Study

**DOI:** 10.2196/37637

**Published:** 2022-09-21

**Authors:** David O Garcia, Luis A Valdez, Benjamin Aceves, Melanie L Bell, Brooke A Rabe, Edgar A Villavicencio, David G Marrero, Forest Melton, Steven P Hooker

**Affiliations:** 1 Department of Health Promotion Sciences Mel and Enid Zuckerman College of Public Health University of Arizona Tucson, AZ United States; 2 Department of Community Health and Prevention Dornsife School of Public Health Drexel University Philadelphia, PA United States; 3 School of Public Health College of Health and Human Services San Diego State University San Diego, CA United States; 4 Department of Epidemiology and Biostatistics Mel and Enid Zuckerman College of Public Health University of Arizona Tucson, AZ United States; 5 Department of Clinical and Translational Sciences College of Medicine University of Arizona Tucson, AZ United States; 6 College of Health and Human Services San Diego State University San Diego, CA United States

**Keywords:** Hispanic, mobile health, mHealth, overweight, obesity, weight loss

## Abstract

**Background:**

Hispanic men have disproportionate rates of overweight and obesity compared with other racial and ethnic subpopulations. However, few weight loss interventions have been developed specifically for this high-risk group. Furthermore, the use of mobile health (mHealth) technologies to support lifestyle behavior changes in weight loss interventions for Hispanic men is largely untested.

**Objective:**

This single-arm pilot study examined the feasibility and acceptability of integrating mHealth technology into a 12-week gender- and culturally sensitive weight loss intervention (GCSWLI) for Hispanic men with overweight and obesity.

**Methods:**

A total of 18 Hispanic men (mean age 38, SD 10.9 years; mean BMI 34.3, SD 5.5 kg/m²; 10/18, 56% Spanish monolingual) received a GCSWLI, including weekly in-person individual sessions, a daily calorie goal, and prescription of ≥225 minutes of moderate-intensity physical activity per week. mHealth technology support included tailored SMS text messaging, behavior self-monitoring support using Fitbit Charge 2, and weight tracking using a Fitbit Aria Wi-Fi Smart Scale. Changes in weight from baseline to 12 weeks were estimated using a paired 2-tailed *t* test. Descriptive analyses characterized the use of Fitbit and smart scales. Semistructured interviews were conducted immediately after intervention to assess the participants’ weight loss experiences and perspectives on mHealth technologies.

**Results:**

Of 18 participants, 16 (89%) completed the 12-week assessments; the overall attrition rate was 11.1%. The mean weight loss at week 12 was −4.7 kg (95% CI 7.1 to −2.4 kg; *P*<.001). Participants wore the Fitbit 71.58% (962/1344) of the intervention days and logged their body weight using the smart scale (410/1344, 30.51% of the intervention days). Participants identified barriers to the use of the technology, such as lack of technological literacy and unreliable internet access for the smart scale.

**Conclusions:**

Although clinically significant weight loss was achieved by integrating mHealth technology into the GCSWLI, adherence to the prescribed use of technology was modest. Addressing barriers to the use of such technologies identified in our work may help to refine an mHealth intervention approach for Hispanic men.

**Trial Registration:**

ClinicalTrials.gov NCT02783521; https://clinicaltrials.gov/ct2/show/NCT02783521

## Introduction

### Background

Hispanics, one of the largest and fastest growing racial and ethnic subpopulations in the United States, comprise 17% of the total US population and are projected to reach 28% by 2060 [1]. In parallel with population growth, the prevalence of obesity continues to increase. Hispanic men, in particular, have the highest age-adjusted prevalence of obesity (BMI≥30 kg/m^2^; 45.7%) when compared with non-Hispanic Black (41.1%) and Hispanic men (44.7%) [2]. Consequently, the incidence of obesity-related diseases such as type 2 diabetes mellitus and nonalcoholic fatty liver disease, which are associated with obesity, is the highest among Hispanic men [3,4]. Thus, it is surprising that despite the growing prevalence of obesity and obesity-related diseases in Hispanic men, weight loss interventions to reduce their body weight are understudied.

Lifestyle interventions have proven to be an effective strategy for implementing chronic disease prevention approaches that are responsive to weight loss [5]. Mobile health (mHealth) technologies, including the use of mobile phones, tablet computers, mobile apps, and wearable devices such as smart watches, are increasingly being used to promote dietary and physical activity behaviors within lifestyle interventions [6,7]. mHealth diminishes access to care barriers for hard-to-reach populations, increases data collection efficiency and accuracy, and provides health care professionals with a means to communicate with participants more frequently in clinical and nonclinical settings [8,9]. Although interventions using mHealth technologies generally have positive results, the methodological standards of evaluated studies are often low [9].

Hispanics are receptive to using mHealth technologies for preventive care services. In particular, SMS text messages have been used as an effective tool for engaging Hispanic participants in research studies [8-11]. A randomized clinical trial by Rosas et al [12,13] demonstrated the effective use of Fitbit devices among Hispanic participants with overweight or obesity, particularly when complemented by a culturally responsive lifestyle intervention. More recently, Rosas [14] found that Hispanic men who chose to attend web-based videoconference sessions lost significantly more weight when compared with a group who chose to watch prerecorded videos on the web. This finding suggests that providing options to accommodate the preferences of Hispanic men for intervention delivery methods can increase engagement in lifestyle interventions [14]. However, the use of mHealth technologies to support lifestyle behavior changes in weight loss interventions for Hispanic men remains largely untested.

### Objectives

Given the rapid growth of the Hispanic population in the United States and the disproportionate burden of preventable chronic diseases faced by this population, effective and sustainable interventions are urgently required. In this context, engaging Hispanic men with mHealth technology that is linguistically and culturally competent, while also meeting their health needs within chronic disease prevention, may be a potential solution. Earlier formative work by our investigative team showed that an mHealth-supported intervention, in combination with face-to-face counseling, may improve engagement and adherence to behavior change recommendations, particularly for physical activity [15-17]. Here, we report the findings from a single-arm pre-post pilot study that examined the feasibility and acceptability of integrating mHealth technology into a 12-week gender- and culturally sensitive weight loss intervention (GCSWLI) targeting Hispanic men with overweight and obesity. We focused on men because they have been shown to be less likely to participate in lifestyle interventions than women [18,19]. In this regard, men, particularly in the Hispanic population, are understudied.

## Methods

### Study Design and Participants

**Figure 1:**
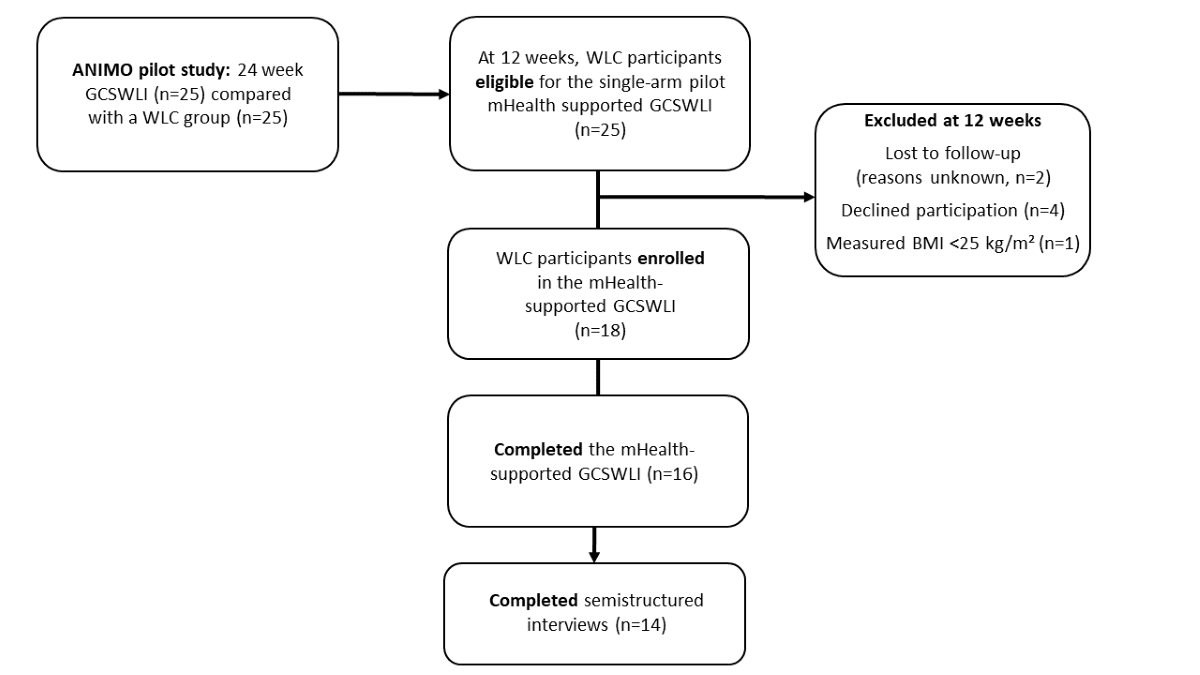
Study flow diagram for the mobile health (mHealth)–supported gender- and culturally sensitive weight loss intervention (GCSWLI). WLC: waitlist control.

Study participants were part of the ANIMO (a Spanish term for motivation or encouragement) pilot study (ClinicalTrials.gov NCT02783521), a 24-week randomized controlled trial that tested the effects of in-person GCSWLI on body weight in Hispanic males (n=25) compared with a waitlist control (WLC) group (n=25) [20,21]. Full details of the ANIMO study design, protocol for the gender- and culturally supported intervention, and outcome findings for the GCSWLI have been published elsewhere [20,21]. Briefly, 98% of the men were of Mexican-origin descent, 58% reported Spanish as their preferred language at home, and 50% were born outside the United States [20,21]. On completion of the initial 12-week GCSWLI, the WLC participants were eligible to receive the mHealth-supported GCSWLI for 12 weeks (Figure 1). To maintain eligibility, men (1) were to be aged 18 to 64 years; (2) had to have a BMI of 25 to 50 kg/m² [22]; (3) had to self-identify as Hispanic; (4) had to have the ability to provide informed consent and complete a health risk assessment before participation; and (5) had to be able to speak, read, and write English or Spanish. Semistructured interviews were conducted after completion of the 12-week intervention to assess participants’ weight loss experiences and perspectives on the potential use of mHealth technologies in future intervention efforts.

### Ethics Approval

All study activities were conducted at the University of Arizona Collaboratory for Metabolic Disease Prevention and Treatment in Tucson, Arizona. Eligible participants were invited to the Collaboratory, where complete details of the study were given in the participant’s preferred language (English or Spanish), and informed consent was obtained. This study was approved by the University of Arizona Institutional Review Board (approval 604536275).

### Description of GCSWLI

Participants met with a trained bilingual, bicultural Hispanic male lifestyle coach in person (one to one) for 30 to 45 minutes once a week for 12 weeks. During these individual counseling sessions, lifestyle coaches assisted participants in setting a daily calorie goal and physical activity goals (progressing to ≥225 minutes of moderate-intensity physical activity per week) and reminded participants to self-monitor in written diaries. The GCSWLI was developed based on the Diabetes Prevention Program lifestyle intervention [23]. Written materials were grounded in social cognitive theory [24] and problem-solving theory [25] and included behavior change techniques shown to be successful in weight loss interventions (eg, self-monitoring, goal setting, and self-efficacy) [26]. Materials were adapted for low literacy (fifth grade reading level) and tailored for gender and cultural factors based on formative work [15-17] and a framework developed by Bernal and Sáez-Santiago [27]. Specifically, we adapted the intervention for gender by including information related to gender role strains (the role of the man in the household) in written materials and discussed how role strains influence health behaviors [20]. This is relevant given that Hispanic culture tends to have traditional and strictly defined gender roles, which may cause differential intervention effects for men and women [28]. To support progress toward goals, participants were required to bring their completed written diaries to counseling sessions. Challenges to meeting diet and physical activity goals were discussed, and lifestyle coaches worked with participants to identify and overcome perceived barriers (eg, strenuous work schedules) to engage in healthy behaviors.

### mHealth Intervention Components

At the conclusion of the 12-week GCSWLI study, specific mHealth components were added to the in-person GCSWLI curriculum used with WLC control participants. Specifically, the use of Fitbit Charge 2, a consumer-wearable physical activity tracker, and a Fitbit Aria Wi-Fi Smart Scale for body weight were included to complement self-monitoring using written diaries. In the first in-person session, all participants were instructed on how to use the Fitbit tools to reduce any barriers related to the introduction of mHealth technology. This included logging into their personal Fitbit dashboard, where the lifestyle coach stored and monitored the data weekly. It was recommended that participants wear the Fitbit Charge 2 during all waking hours and weigh themselves daily using the Aria smart scale. The Fitbit data were downloaded during weekly in-person sessions and discussed with the participants by the lifestyle coach. A total of 2 weekly SMS text messages were sent to participants throughout the following week by a lifestyle coach, tailored to the participants’ needs based on observations from the Fitbit tools and self-reported barriers during the weekly in-person sessions. All intervention strategies from the original GCSWLI curriculum were maintained to ensure that participants had the opportunity to lose weight, independent of mHealth use.

### Quantitative Outcome Measures

Clinical assessments included anthropometrics, cardiometabolic measures, and self-reported diet and physical activity behaviors at baseline and 12 weeks. Acculturation was measured at baseline, and treatment satisfaction was measured at the completion of the study.

### Anthropometrics and Cardiometabolic Measures

Height (cm) was measured using a wall-mounted stadiometer, and weight (kg) was measured using a calibrated digital scale (Seca 876). Waist circumference (cm) was measured using a Gulick tape measure, and body composition was measured using whole-body dual-energy x-ray absorptiometry (Lunar Prodigy; Lunar). A trained phlebotomist collected fasting blood samples (venipuncture, 25 mL) using an approved protocol for examining the following cardiometabolic measures: (1) a metabolic liver panel (alanine transaminase and aspartate transaminase), (2) a lipid panel (total cholesterol, high-density lipoprotein, low-density lipoprotein, and triglycerides), (3) high-sensitivity C-reactive protein, and (4) fasting glucose and hemoglobin A_1c_.

### Self-reported Dietary Intake and Physical Activity

Dietary intake was assessed using the Southwestern Food Frequency Questionnaire (SWFFQ) [29]. The SWFFQ is a bilingual food frequency questionnaire adapted from the Arizona Food Frequency Questionnaire. It includes more than 150 food items, such as corn and flour tortillas, nopalitos, machaca, and chorizo, which are often consumed by individuals in southwest United States. The SWFFQ uses relevant Mexican names for food items (eg, *naranja,* not *china*, for *orange*) and asks participants to describe their average use (ranging from 3 or more times daily to rarely or never) and portion size (small, medium, or large) for each food item. Data retrieved from the SWFFQ allowed for the calculation of total daily energy intake and percentage of daily energy intake [30]. Physical activity was assessed using the validated Global Physical Activity Questionnaire [31], which asked participants to describe their activity at work, travel to and from places, recreational activities, and sedentary behavior. The questionnaire is available in both English and Spanish and provides the duration (minutes per week) and intensity of physical activity (moderate to vigorous).

### Acculturation Levels and Treatment Satisfaction

Acculturation affects attitudes, perceptions, and behaviors surrounding health-related activities and outcomes [32]. Therefore, we assessed the level of acculturation related to language, ethnic identity, and ethnic interaction in the study participants using the validated Acculturation Rating Scale for Mexican Americans-II [33], which is available in both English and Spanish. Treatment satisfaction was assessed at study completion (12 weeks) using 4 questions rated on a Likert scale, with higher scores indicating greater program favorability [34]. Questions prompted participants to rate their overall satisfaction with their progress toward changing dietary and physical activity behaviors for weight management and asked participants whether they would recommend the program to others [35].

### Qualitative Measures: Semistructured Interviews

All participants were invited to participate in individual postintervention interviews. The interview protocol was devised to elicit information about (1) participant satisfaction with the intervention, (2) adequacy of cultural and gender intervention adaptation, (3) strategies for improved intervention engagement, (4) adequacy of physical activity and dietary recommendations, and importantly, (5) perspectives on the integration of mHealth technology as a component in weight management interventions. Written and verbal consent was obtained from each willing participant, and an additional US $25 was offered as compensation. All interviews were conducted by each participant’s respective lifestyle coach in their preferred language and were audio recorded to facilitate data interpretation. To minimize bias, all interviewers carefully explained that the participants were free and under no pressure to express their views and that the focus of the interviews was to learn their perceptions to help inform the future development of similar intervention programs.

### Quantitative Statistical Analyses

Descriptive statistics were calculated for all the variables at baseline. To explore differences in baseline variables between the enrolled group and the group that did not enroll in the mHealth pilot study, we conducted 2-tailed *t* tests and chi-square tests on continuous and categorical demographic and anthropometric variables, respectively. Study attrition and process measures, including attendance at individual sessions, self-monitoring adherence, use of mHealth technology support, and treatment satisfaction, were examined using descriptive analyses. Attrition was calculated for those who did not complete 12 weeks of treatment. One-sample 2-tailed *t* tests were used to estimate the mean changes in body weight and anthropometric measures, as well as the secondary outcomes of eating behaviors, physical activity levels, and cardiometabolic biomarkers. Participants with missing blood or weight data at week 12 were excluded from the analysis. Statistical analyses were performed using SAS (version 9.4; SAS Institute Inc). *P* values of <.05 were considered statistically significant.

### Qualitative Data Analyses

All individual interviews were digitally recorded, transcribed verbatim in their respective languages (Spanish or English), and coded using NVivo (version 10, QSR International) qualitative analysis software [36]. For the purposes of this study, we analyzed only qualitative data evaluating the inclusion of mHealth technology in the weight loss intervention. As such, all interviews that elicited statements including mentions of the use of mHealth technology in the intervention were compartmentalized and included in the thematic analysis. The purpose of this analysis was to characterize participants’ opinions and perspectives regarding the use of mHealth technology as a component in weight management interventions for Hispanic men.

## Results

### Overview

On completion of the initial 12-week GCSWLI, WLC participants (n=25) were offered the mHealth-supported intervention. Overall, 2 patients were lost to follow-up for unknown reasons, 4 declined participation, and 1 individual had a measured BMI 25 kg/m^2^ before the start of the treatment and was deemed ineligible (Figure 1). A total of 18 participants were younger (38 vs 49 years; *P*=.04) and weighed more (105 vs 90 kg; *P*=.02) than those who elected not to participate. Other demographic characteristics and acculturation levels did not differ between groups (Table 1).

Overall, an average of 75% (SD 27%) of the individual sessions were attended throughout the 12-week treatment period. Of the 18 participants, 2 (11%) attended <50% of the sessions, 6 (33%) attended 50% to 75% of the sessions, and 10 (56%) attended >75% of the sessions. Of the 18 participants, 16 (89%) completed the intervention, with an overall attrition rate of 11.1%. Self-monitoring adherence and mHealth technology use at the follow-up are shown in Table 2.

On average, each person completed 6.2 weekly written diaries; 51.93% (698/1344) of the total diaries were completed. Diet was recorded on average 1.7 days per week, with an average of 1260 kcal reported each day. Participants reported physical activity an average of 1.4 days per week, with a total of 78 minutes of weekly exercise, and they recorded their weight on an average of 1.8 days per week. Activity monitors were used 5.1 days per week, and weight was reported using wireless scales 3.2 days per week. Overall, participants wore the Fitbit 71.6% of the intervention days and weighed themselves using the smart scale for 30.5% of the days they spent in the intervention. The participants were highly satisfied with the weight management program, and 100% (15/15) of the participants indicated that they were likely to recommend the program to others. Satisfaction with overall progress toward weight loss was positive, with progress in changing dietary habits ranking the highest (Table 3).

The pre- and postweight and cardiometabolic measures are shown in Table 4. Weights were significantly reduced by an average of −4.7 kg (95% CI −7.1 to −2.4 kg; *P*<.001); 7 individuals (44%) achieved clinically significant weight loss of ≥5%. In addition, significant changes in BMI by −1.6 kg/m^2^ (−2.4 to −0.8 kg/m^2^), body fat by −1.97% (−2.98% to −0.96%), and waist circumference by −5.1 cm (−6.8 to −3.4 cm) were observed after the 12-week intervention (*P*<.001). Physical activity increased, and the average caloric intake decreased, but the changes were not statistically significant. Significant decreases in cardiometabolic measures were found in hemoglobin A_1c_ (*P*=.02), alanine transaminase (*P*=.04), and total cholesterol (*P*=.007).

**Table 1:** Demographic and baseline characteristics of participants in a mobile health (mHealth)–supported gender- and culturally sensitive weight loss intervention.

			mHealth participants (n=18)		Did not enroll (n=7)		*P* value
Age (years), mean (SD)			38.0 (10.9)		48.9 (12.1)		.04
BMI (kg/m^2^), mean (SD)			34.3 (5.5)		30.3 (3.6)^a^		.14
Weight (kg), mean (SD)			104.6 (20.8)		90.0 (6.3)^a^		.02
Waist circumference (cm), mean (SD)			114.1 (14.6)		107.5 (5.7)^a^		.34
Employed, n (%)			12 (66)		6 (85)		.63
**Income (US $), n (%)**							.99
	<30,000	13 (72)		5 (71)			
	30,000-60,000	4 (22)		2 (29)			
	>60,000	1 (6)		0 (0)			
**Education, n (%)**							.99
	Less than high school	6 (33)		2 (29)			
	High school or General Educational Development	5 (28)		2 (29)			
	Greater than high school	7 (39)		3 (43)			
Married or lives with a domestic partner, n (%)			14 (78)		7 (100)		.30
US born, n (%)			10 (56)		1 (14)		.09
**Heritage, n (%)**							.53
	Mexican	10 (56)		6 (86)			
	Mexican American	7 (39)		1 (14)			
	Puerto Rican	1 (6)		0 (0)			
**Primary language spoken at home, n (%)**							.39
	Spanish	10 (56)		6 (86)			
	English	6 (33)		1 (14)			
	Both equally	2 (11)		0 (0)			
Mexican Orientation Subscale of ARSMA-II^b^, mean (SD)			65.6 (16.7)		73.6 (8.2)		.24
**Acculturation level (ARSMA-II), n (%)**							.79
	Very Mexican oriented	7 (39)		5 (71)			
	Mexican oriented to approximately balanced to oriented bicultural	6 (33)		1 (14)			
	Slightly Anglo bicultural	3 (17)		1 (14)			
	Strongly Anglo oriented	1 (6)		0 (0)			
	Very assimilated	1 (6)		0 (0)			

^a^A total of 2 individuals dropped out owing to their participation in the waitlist control.

^b^ARSMA-II: Acculturation Rating Scale for Mexican Americans-II.

**Table 2:** Attendance, self-monitoring adherence, and mobile health (mHealth) technology use in a gender- and culturally sensitive weight loss intervention (N=18).

Characteristics				Values
**Overall percentage of attendance at individual sessions, mean (SD)**				75.0 (27.0)
	Attended <50% of the sessions, n (%)		2 (11)	
	Attended 50%-75% of the sessions, n (%)		6 (33)	
	Attended >75% of the sessions, n (%)		10 (56)	
**Self-reported measures^a^**				
	Diaries completed, n (%)		112 (52)	
	Diaries completed per person, mean (SD)		6.2 (4.2)	
	Diet days recorded (days per week), mean (SD)		1.7 (2.8)	
	Self-reported calorie intake (kcal per day), mean (SD)		1260 (566)	
	**Self-reported physical activity** **, mean (SD)**			
		Days per week	1.4 (2.0)	
		Minutes per week	78.0 (63.1)	
	Self-weighed (days per week), mean (SD)		1.8 (2.9)	
**mHealth measures, mean (SD)**				
	Activity monitor use (days per week)		5.1 (2.0)	
	Activity monitor use during sleep (days per week)		2.2 (2.4)	
	Diet days recorded via web (days per week)		0.4 (1.3)	
	Weight reported using wireless scale (days per week)		3.2 (3.2)	

^a^Data obtained from paper diary logging.

**Table 3:** Participant responses to treatment satisfaction survey (for 15 of 16 completers).

Participant responses		Values
**How satisfied are you overall with the weight management program?^a^ n (%)**		
	Somewhat satisfied	1 (7)
	Very satisfied	13 (87)
	Missing response	1 (7)
Would you recommend the weight management program you received to others?^b^ (definitely would), n (%)		15 (100)
Given the effort you put into following the weight management program, how satisfied are you with your overall progress over the past 12 weeks?^c^ median (IQR)		3.0 (2.5-4.0)
**Given the effort you put into following the weight management program for the past 12 weeks, how satisfied are you overall with your progress on?^c^ median (IQR)**		
	Changing your weight	3.0 (3.0-4.0)
	Changing your dietary habits	3.0 (2.5-4.0)
	Changing your physical activity habits	3.0 (2.0-4.0)

^a^1=very dissatisfied and 4=very satisfied.

^b^1=definitely not and 4=definitely would.

^c^4=very dissatisfied and 4=very satisfied.

**Table 4:** Outcomes before and after the mobile health intervention and estimated mean change from baseline.

			Baseline (n=18), mean (SD)		Week 12 (n=16), mean (SD)		Change from baseline, mean (95% CI)		*P* value
Weight (kg)			104.6 (20.8)		100.3 (21.6)		−4.7 (−7.1 to −2.4)		<.001
BMI (kg/m^2^)			34.3 (5.5)		32.7 (5.9)		−1.6 (−2.4 to −0.8)		<.001
Percentage weight change			—^a^		−4.8 (4.5)		−4.8 (−7.0 to −2.0)		<.001
Percentage body fat			37.3 (3.6)		35.3 (4.8)		−1.97 (−2.98 to −0.96)		<.001
Waist circumference (cm)			114.1 (14.6)		109.5 (14.3)		−5.1 (−6.8 to −3.4)		<.001
Leisure-time physical activity (min/week)			87.2 (100.6)		139.4 (170.8)		60.0 (−44.6 to 164.6)		.24
Average caloric intake (kcal/day)			3102 (3745)		1754 (1329)		−1196 (−2647 to 254)		.11
**Cardiometabolic measures**									
	Hemoglobin A_1c_ (%)	5.6 (0.8)		5.5 (0.6)		−0.22 (−0.41 to −0.03)		.02	
	Glucose (mg/100 mL)	105.3 (28.4)		105.4 (32.8)		−2.31 (−9.26 to 4.64)		.49	
	Alanine transaminase (U/L)	53.6 (52.7)		39.6 (28.1)		−16.9 (−32.5 to −1.23)		.04	
	Aspartate transaminase (U/L)	28.9 (19.3)		24.9 (9.6)		−5.3 (−11.7 to 1.04)		.10	
	Total cholesterol (mg/100 mL)	185.7 (40.4)		171.4 (37.5)		−11.8 (−19.9 to −3.7)		.007	
	High-density lipoprotein cholesterol (mg/100 mL)	40.2 (5.6)		39.3 (6.9)		−1.0 (−4.2 to 2.2)		.51	
	Low-density lipoprotein cholesterol (mg/100 mL)	113.7 (34.3)		104.8 (31.8)		−5.6 (−12.0 to 0.80)		.08	
	Triglycerides (mg/100 mL)	158.8 (82.2)		136.4 (71.5)		−25.6 (−52.0 to 0.9)		.06	
	High-sensitivity C-reactive protein (mg/100 mL)	3.4 (2.9)		2.5 (2.5)		−0.83 (−2.37 to 0.70)		.26	

^a^Not available.

### Participant Experience With mHealth Components

Of the 16 participants who completed the intervention, 14 (88%) agreed to participate in an individual interview after the completion of the 12-week intervention. A total of 4 overarching themes emerged during these interviews regarding participants’ perceptions of the integration of mHealth technology into a weight management intervention.

When asked to reflect on their use of Fitbit activity and weight trackers, participants expressed positive feedback and no concerns about the security or privacy of the e-supported data were reported. The participants explained that mHealth technology was at times easier to use than the written diary self-monitoring method. This, in turn, facilitated the seamless integration of self-monitoring into daily lifestyle behavior changes. For example, a participant commented the following:

And of course the best part that I really enjoyed were the technological tools, the Fitbit the scale things like that the Fitbit app that made a huge world of difference. You know it became part of my life and now I use it all the time to count my steps look at information look at data look at how I sleep and how much exercise I got that day it’s great it’s a great thing to have.

Participants also expressed that using the Fitbit wearable tracking device facilitated accountability to daily goals. Specifically, the participants expressed that it was easy to see when daily goals were not being met and that they enjoyed the positive feedback they received when goals were met. A participant stated the following:

[I liked the Fitbit] because I can have a record of whether or not I walk, you understand me? Instead of forgetting...and when I do wrap up a night, it will tell me, “hey great job! You walked like 10, 20, 25 minutes” and I pay attention to that. So that helps.

Another participant commented on the utility of activity trackers to standardize step count goals, explaining as follows:

I like [the Fitbit] because, how do I explain? I can at least say that 10,000 steps is like the bottom line, right?...So, when I realize that I have not reached 3,000 steps for two or three days I really start paying attention.

Conversely, some participants expressed concerns about the use of technology. For some, reliable internet connectivity was a barrier to the use of supplemental technology. The Fitbit scale requires a reliable internet connection to sync and upload data, which some participants did not have access to. A few men also explained that an additional technology-centered component was an added burden instead of a supplemental benefit of the intervention. This was particularly the case with older participants, who reported less technology literacy because technology was not already integrated into their daily lives. A participant described the following:

I think that was helpful. I don’t know that the Fitbit thing would have been very helpful to me I’m not a real tech-oriented guy I don’t want to sync up this and that enter info, yeah so I’m not, I’m not into that so I don’t know if that would have helped me, but other things did that’s for sure.

## Discussion

### Principal Findings

Hispanic men are part of the largest racial and ethnic subpopulation in the United States and are disproportionally affected by obesity-related chronic diseases [37]. Although broad systemic sociopolitical and sociocultural factors have an undeniable and ubiquitous influence on this burden, disparities are also partly owing to the lack of tailored culturally and linguistically responsive programs. The overarching objective of this pilot study was to examine the feasibility and acceptability of integrating mHealth technology into a 12-week GCSWLI for Hispanic men with overweight and obesity. The findings of this work help elucidate the role that mHealth technologies can play in supporting the delivery of preventive and interventional care to at-risk and hard-to-reach groups, including Hispanic men of Mexican origin.

### Comparison With Prior Work

The findings of this study yielded promising improvements in body composition, including changes in body weight, body fat percentage, and waist circumference. Recent literature assessing body composition outcomes has yielded only modest results in other Hispanic subpopulations and has been largely limited to enrollment of women [38,39]. In fact, only a few studies have focused solely on Hispanic men. In the previously mentioned ANIMO study, where mHealth technology was not included, the mean weight loss in the GCSWLI at week 12 was −6.3 kg (95% CI −8.1 to −4.4 kg), and it was maintained at week 24 (−6.4 kg, 95% CI −8.6 to −4.3 kg) [21]. More recently, Rosas et al [14] conducted a study focusing on a choice-based technology-mediated lifestyle intervention for 200 Latino men. It compared 3 weight loss intervention options (videoconference, web-based videos, and in person) based on participants’ choices. Overall, 41% (82/200) chose the in-person group, 31% (62/200) chose the web-based video group, and 28% (56/200) chose the videoconference group. The authors found that participants who initially chose the videoconference and in-person group sessions lost significantly more weight at 6 months (mean −3.9, SD 6.1 kg for videoconference and mean −4.3, SD 5.3 kg for in-person sessions) compared with web-based videos (mean −0.3, SD 3.7 kg). Similar weight loss was observed for our mHealth-supported GCSWLI at week 12 (−4.7 kg, 95% CI −7.1 to −2.4 kg). Notably, attendance was high across all 3 studies, independent of the intervention delivery method. For example, attendance at individual counseling sessions for ANIMO was 77% and was 75% (144/192) for this mHealth study. Rosas et al [14] observed that the mean number of sessions attended out of 12 weeks was 10.9 (SD 2.6) for the videoconference sessions and 9.7 (SD 4.3) for in-person sessions. Continued efforts to examine which aspects of these studies, including delivery options and which intervention components (eg, mHealth) influence study effectiveness, are warranted.

Regarding feasibility and acceptability, our mHealth intervention was well received and produced a retention rate of 88.9%. In general, weight loss interventions in Hispanic adults have previously reported lower retention rates; however, they did not include mHealth components [40]. Rosas et al [14] observed a retention rate of 96.9%, suggesting that technology-mediated strategies may support retention efforts [14]. Our findings suggest moderate adherence to mHealth technology prescriptions, as the Fitbit Charge 2 wearable activity monitor was used 71.58% (962/1344) of the intervention days, and the Fitbit Aria Wi-Fi scale was used 30.51% (410/1344) of the intervention days. However, the completion rate of diaries (698/1344, 51.93%) was lower than that in the ANIMO study (70%) [21]. This suggests that adding mHealth technology decreased adherence to self-monitoring behaviors. For example, diet behaviors were reported only 1.7 days per week, with an average of 1260 kcal reported each day, which was likely underreported. However, our findings align with a recent systematic review that suggests wearable tracking devices as part of weight loss interventions appear to be feasible when incorporated in short-term (<6 months) comprehensive weight loss programs [41]. We found that using mHealth tools was well received in a 12-week weight loss intervention for Hispanic men. Specifically, the qualitative findings suggested that participants’ perceptions on the use of mHealth technology specific to this study were acceptable and facilitated accountability to lifestyle behaviors during the study.

Hispanic men experienced high levels of overall satisfaction with this intervention modality expressing that they would recommend participation to other Hispanic men. This is in agreement with the findings of Wang et al [9] who confirmed that wearable technologies and apps appear to have high efficacy when used in the context of medical care and coaching for weight loss. Nevertheless, a few participants cited internet connection as a barrier to the use of the Aria Scale for weighing themselves daily. This may have affected our findings, as daily weighing has been shown to improve weight loss and adoption of weight control behaviors [42]. This finding aligns with other studies that have cited internet accessibility as a barrier to intervention delivery [8]. Mayberry et al [8] reported in their findings that internet access remains unavailable to multiple segments of disadvantaged or vulnerable populations, including people with low socioeconomic status, members of racial and ethnic groups, and persons with limited health literacy or numeracy skills. Future research focusing on the incorporation of technology tracking devices into comprehensive weight loss interventions should prioritize the provision of reliable internet connectivity to participants. This can ensure that these devices fulfill their purpose of complementing participants’ efforts for behavior change instead of becoming an obstacle to their goals.

### Limitations

Although this study has many strengths, we must acknowledge some limitations, including a small study sample, a short time frame, and a 1-group pre-post design that did not allow direct comparison with a control group. Therefore, we cannot disentangle which mHealth components were most supportive of weight loss, and the results must be interpreted with caution. Furthermore, although most individuals achieved weight loss, we did not observe statistically significant changes in diet or physical activity. Recall bias should be considered as a hindering factor when self-reporting techniques are involved. However, the investigative team attempted to minimize recall bias by reviewing the questionnaires in person for each participant. Furthermore, given that all study participants came from Tucson, Arizona, and were largely of Mexican-origin descent apart from one individual, this decreases the generalizability of findings and adaptability to Hispanic men in other areas. There is also a possibility that increased adherence to mHealth technology could have been due to the novelty of using the Fitbit wearable technology and gaining access to high-level health care tools such as dual-energy x-ray absorptiometry scan and cardiometabolic measures. Future research in this area should explore the impact of this intervention modality beyond 12 weeks to explore the progression of healthy behavior continuity and adherence levels to wearable technology in the long term. In addition, interventions should focus on underrepresented and underresourced subpopulations that are disproportionately affected by health inequalities and have unequal access to high-quality health services and information.

### Conclusions

The integration of mHealth technology into our 12-week GCSWLI appeared to be feasible and widely accepted by study participants. Although the use of wearable technology was modest, Hispanic men achieved clinically meaningful weight loss at the end of the intervention. This pilot study contributes to the growing literature on health promotion and mHealth technologies to aid the adoption of healthy lifestyle behavior changes implemented through weight loss interventions for Hispanic men. Research efforts centered on weight management, and mHealth should consider culturally adapted frameworks that address barriers hindering the awareness and maintenance of healthy lifestyles. The use of mHealth holds promise in lifestyle interventions for Hispanic men, making healthy living opportunities more practical and achievable for communities that have historically lacked the benefits of this type of technology.

## Abbreviations

GCSWLI: gender- and culturally sensitive weight loss intervention
mHealth: mobile health
SWFFQ: Southwestern Food Frequency Questionnaire
WLC: waitlist control
